# Identifying G6PC3 as a Potential Key Molecule in Hypoxic Glucose Metabolism of Glioblastoma Derived from the Depiction of ^18^F-Fluoromisonidazole and ^18^F-Fluorodeoxyglucose Positron Emission Tomography

**DOI:** 10.1155/2024/2973407

**Published:** 2024-02-28

**Authors:** Michinari Okamoto, Shigeru Yamaguchi, Ryosuke Sawaya, Sumire Echizenya, Yukitomo Ishi, Sadahiro Kaneko, Hiroaki Motegi, Takuya Toyonaga, Kenji Hirata, Miki Fujimura

**Affiliations:** ^1^Department of Neurosurgery, Hokkaido University Graduate School of Medicine, North 15 West 7, Kita-ku, Sapporo 060-8638, Japan; ^2^Department of Diagnostic Imaging, Hokkaido University Graduate School of Medicine, North 15 West 7, Kita-ku, Sapporo 060-8638, Japan

## Abstract

**Purpose:**

Glioblastoma is the most aggressive primary brain tumor, characterized by its distinctive intratumoral hypoxia. Sequential preoperative examinations using fluorine-18-fluoromisonidazole (^18^F-FMISO) and fluorine-18-fluorodeoxyglucose (^18^F-FDG) positron emission tomography (PET) could depict the degree of glucose metabolism with hypoxic condition. However, molecular mechanism of glucose metabolism under hypoxia in glioblastoma has been unclear. The aim of this study was to identify the key molecules of hypoxic glucose metabolism.

**Methods:**

Using surgically obtained specimens, gene expressions associated with glucose metabolism were analyzed in patients with glioblastoma (*n* = 33) who underwent preoperative ^18^F-FMISO and ^18^F-FDG PET to identify affected molecules according to hypoxic condition. Tumor *in vivo* metabolic activities were semiquantitatively evaluated by lesion-normal tissue ratio (LNR). Protein expression was confirmed by immunofluorescence staining. To evaluate prognostic value, relationship between gene expression and overall survival was explored in another independent nonoverlapping clinical cohort (*n* = 17) and validated by The Cancer Genome Atlas (TCGA) database (*n* = 167).

**Results:**

Among the genes involving glucose metabolic pathway, mRNA expression of *glucose-6-phosphatase 3* (*G6PC3*) correlated with ^18^F-FDG LNR (*P* = 0.03). In addition, *G6PC3* mRNA expression in ^18^F-FMISO high-accumulated glioblastomas was significantly higher than that in ^18^F-FMISO low-accumulated glioblastomas (*P* < 0.01). Protein expression of G6PC3 was consistent with mRNA expression, which was confirmed by immunofluorescence analysis. These findings indicated that the G6PC3 expression might be facilitated by hypoxic condition in glioblastomas. Next, we investigated the clinical relevance of *G6PC3* in terms of prognosis. Among the glioblastoma patients who received gross total resection, mRNA expressions of *G6PC3* in the patients with poor prognosis (less than 1-year survival) were significantly higher than that in the patients who survive more than 3 years. Moreover, high mRNA expression of *G6PC3* was associated with poor overall survival in glioblastoma, as validated by TCGA database.

**Conclusion:**

G6PC3 was affluently expressed in glioblastoma tissues with coincidentally high ^18^F-FDG and ^18^F-FMISO accumulation. Further, it might work as a prognostic biomarker of glioblastoma. Therefore, G6PC3 is a potential key molecule of glucose metabolism under hypoxia in glioblastoma.

## 1. Introduction

Glioblastoma is the most aggressive tumor among diffuse gliomas and is defined as grade IV according to the 2016 World Health Organization (WHO) classification [1]. Recently, the prognosis of patients with glioblastoma has been gradually improved, owing to the development of image-guided surgery, radiotherapy, and new chemotherapeutic drugs; however, the 2-year survival rate remains at 43% only [2]. As one of the mechanisms that make tumors more resistant to therapy, hypoxia, a condition where oxygen requirements in a tissue exceed its supply, is closely related to the characteristics of malignant tumors [3]. Consistent with other malignant neoplasms, glioblastoma frequently occurs in severe hypoxia [4–9]. Since fluorine-18^−^-fluoromisonidazole (^18^F-FMISO) was developed as hypoxic radio-labeled tracer using positron emission tomography (PET), ^18^F-FMISO PET has been useful in detecting hypoxic parts of brain tumors [7].

**Figure 1:**
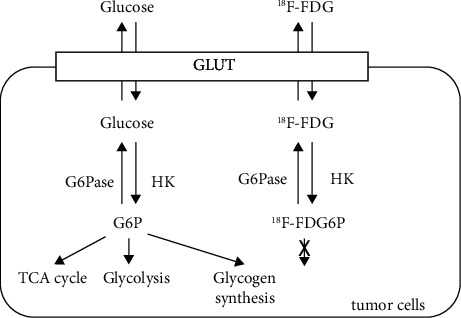
Schematic for the metabolic trapping of fluorodeoxyglucose in tumor cells. Fluorine-18-fluorodeoxyglucose (^18^F-FDG) is accumulated in tumor cells via glucose transporter and is phosphorylated by hexokinase (HK). Glucose-6-phosphatase counteracts HK phosphorylation. Glucose-6-phosphate is consumed for energy production although FDG is not metabolized. G6P: glucose-6-phosphate; G6Pase: glucose-6-phosphatase; HK: hexokinase; TCA: tricarboxylic acid; ^18^F-FDG: fluorine-18-fluorodeoxyglucose; ^18^F-FDG6P: fluorine-18-fluorodeoxyglucose-6-phosphate.

Fluorine-18-fluorodeoxyglucose (^18^F-FDG) is a common radiotracer that can visualize intracellular glucose metabolism. Since glycolysis is highly activated in cancer cells (Warburg effects), ^18^F-FDG uptake is increased in cancer cells. Hence, ^18^F-FDG PET has been useful in detecting cancer cells [10]. Similarly in glioma, ^18^F-FDG is accumulated in proportion to tumor malignancy [11]. ^18^F-FDG uptake is determined by multiple factors, such as glucose transporter (GLUT), hexokinase (HK), or glucose-6-phosphatase (G6Pase) based on “metabolic trapping” as an ^18^F-FDG kinetic principle [12, 13]. The molecular mechanisms of glucose metabolism in ^18^F-FDG PET were reportedly associated with ^18^F-FDG uptake by GLUT and metabolism by HK and G6Pase in Figure 1 [14]. As GLUT and HK play important roles in glycolysis, G6Pase acts as a gluconeogenic enzyme and is categorized into three subtypes: glucose-6-phosphatase catalytic unit (G6PC) 1–3 [15, 16].

We previously showed that semiquantitative evaluation by ^18^F-FDG and ^18^F-FMISO PET could demonstrate metabolically active hypoxic volume, a significant predictor for progression-free survival and overall survival (OS) in glioblastoma [9]. However, the underlying mechanism remains unclear. Based on the fact that hypoxia can induce glycolysis [17], we hypothesized that any glycolysis-associated molecules induced by hypoxia might influence the prognosis of patients with glioblastoma. We aimed to identify the key molecules associated with active glucose metabolism under hypoxia, and the expression of these glucose- and hypoxic-related genes in glioblastoma tissues obtained from the patients who underwent preoperative ^18^F-FDG and ^18^F-FMISO PET was analyzed. Then, we investigate whether the identified molecule is associated with the prognosis of patients with glioblastoma.

## 2. Materials and Methods

### 2.1. Surgical Excision of Glioblastoma Specimens

Patients who suffered from glioblastoma and underwent both ^18^F-FMISO and ^18^F-FDG PET preoperatively between 2008 and 2016 in Hokkaido University Hospital (Sapporo, Japan) were included. The surgical procedure was performed in the same manner as described previously [18]. We used an intraoperative neuronavigation system (Stealth Station S7®, Medtronic, Louisville, CO, USA). Gadolinium-enhanced T1-weighted imaging (Gd-T1WI) with three-dimensional spoiled gradient echo (3D-SPGR) magnetic resonance and PET images were transferred to the navigation system. The surgical procedure was either biopsy or tumor resection. In both procedures, the tissue showing the highest FDG tracer uptake was carefully resected and cryopreserved at −80°C.

### 2.2. Sample Selection and Genetic Analysis of IDH Mutation

To perform the gene expression analysis of glucose metabolism and related molecules, surgical specimens obtained from the patients were selected according to the following criteria: (1) obtained during primary tumor resection without any adjuvant therapy, including anticancer chemotherapy or radiation therapy, and (2) histologically diagnosed as glioblastoma based on the WHO classification [1]. DNA/RNA extraction from the frozen tumor tissue and analysis of the mutational status of *isocitrate dehydrogenase (IDH) 1/2* were performed as previously described [19].

### 2.3. Image Acquisition and Reconstruction

For each patient, ^18^F-FDG and ^18^F-FMISO PET were performed in random order within 1 week. PET imaging protocols for both ^18^F-FDG and ^18^F-FMISO were consistent with our previous studies [7, 9]. PET scans were conducted using one of following three PET scanners: an ECAT EXACT HR+ PET scanner (Siemens, Munich, Germany), a Biograph 64 PET computed tomography (CT) scanner (Siemens, Munich, Germany), and Gemini TF64 PET-CT scanner (Philips, Amsterdam, Netherlands). All scanners were operated in a three-dimensional reconstruction mode. On the ECAT EXACT HR+ PET scanner, the emission acquisition was performed after a 3 min transmission scan with a ^68^Ge/^68^Ga retractable line source. On the Biograph 64 PET/CT and Gemini TF64 PET-CT scanners, CT scanning was performed after an emission acquisition. Transmission images from HR+ and CT images from Biograph 64 PET/CT and Gemini TF64 PET-CT were used to estimate attenuation maps. Attenuation-corrected radioactivity images were reconstructed using the filtered back-projection (ECAT EXACT HR+ and Biograph 64) or the ordered subset expectation maximization method (Gemini TF64) with a Hann filter of 4 mm full width at half maximum. For ^18^F-FDG PET, patients had fasted for >6 h before the scheduled tracer injection. ^18^F-FDG was intravenously injected, and 10 min emission scanning was performed at 1.23 ± 0.3 h post-tracer injection. The actual injected dose was 323.3 ± 90.6 MBq. For ^18^F-FMISO PET, 10 min static PET scanning was performed at 4.06 ± 0.3 h post-tracer injection, according to our previously investigation [20]. The actual injected dose was 413.9 ± 28.2 MBq.

### 2.4. Imaging Analysis

The lesion-to-normal cerebellum ratio (LNR) was calculated for semiquantitative analysis as previously described [8]; the maximum standardized uptake value (SUV) of the tumor was divided by the average cerebellar SUV and LNR.

### 2.5. Quantitative Reverse Transcription-Polymerase Chain Reaction (qRT-PCR) Analysis

As a control reference, two sets of commercially available human brain total RNA were obtained (Life Technologies; Clontech). The glioma cDNA was synthesized from 1 mg of the total RNA using the PrimeScript™ II 1st Strand cDNA Synthesis Kit (Takara Biotechnology Co., Ltd., Kusatsu, Japan). Several genes associated with hypoxia and glucose metabolism were selected, including *GLUT1*, *GLUT3*, *G6PC1*, *G6PC2*, *G6PC3*, *HK1*, *HK2*, *vascular endothelial growth factor* (*VEGF*), and *proliferation cell nuclear antigen* (*PCNA*). The oligonucleotide primers are listed in Table S1. qRT-PCR analysis was performed using FastStart Essential DNA Green Master with LightCycler 96 (Roche Diagnostics, Basel, Switzerland), and the PCR product specificities were confirmed by melt curve analysis. PCR experiments were run in triplicate. The relative target gene mRNA expression levels compared to 18S rRNA were measured by qPCR using the 2^−*ΔΔ*CT^ method [21]. The relationship between mRNA expression and LNR of ^18^F-FDG PET was explored. Moreover, each mRNA expression was evaluated in two groups of specimens categorized by the degree of hypoxia in a 2.0 threshold of FMISO LNR, as has been described previously [22].

### 2.6. Immunofluorescence Analysis

To evaluate the protein expression of G6PC3 and GLUT1, triple staining was performed in a glioblastoma sample with antibodies against G6PC3 (1 : 100, ab221647, Abcam plc, Cambridge, UK) and GLUT1 (1 : 400, 66290-1-IG, Proteintech, Manchester, UK). A formalin-fixed paraffin-embedded (FFPE) block was used for antigen retrieval as previously described [23] and then blocked with Block Ace (UKB80, DS Pharma Biomedical, Osaka, Japan). Diluted with 1%BSA-PBC were added followed by overnight incubation at 4°C. As secondary antibodies, Alexa Fluor goat anti-mouse-488 (1 : 400, A11001, Invitrogen, Massachusetts, USA) and goat anti-rabbit-594 (1 : 400, A11012, Invitrogen,). All nuclei were stained with ProLong Diamond antifade reagent (P36962, Invitrogen, Massachusetts, USA). All images of Immunofluorescence staining was obtained using a fluorescent microscope (BZ-X710, KEYENCE, Osaka, Japan).

### 2.7. Association between Candidate Gene Expression and Survival

To analyze the association between candidate gene expression and prognosis in glioblastoma, we subjectively selected patients with glioblastoma independent of the discovery cohort. Among the patients with glioblastoma who underwent gross total resection of primary tumors prior to the standard treatment [24], eight patients with >3 years of survival after the primary surgery were selected as the good prognosis group and 9 patients with <1 year of survival as the poor prognosis group. RNA extraction from each frozen tumor tissues and gene expressions of candidate molecule were performed as the same manner as discovery cohort.

### 2.8. Data Mining in TCGA

Clinical information, gene expression, and gene mutation status were obtained from The Cancer Genome Atlas (TCGA, https://portal.gdc.cancer.gov/) database for patients with glioblastoma. Patients aged <16 years at the time of diagnosis and those without survival data were excluded. The raw count data of RNA-seq were obtained from TCGA and then normalized using the edgeR package (version 3.26.1) in R (version 3.5.3). The OS of patients with glioblastoma in TCGA was evaluated according to mRNA expression of candidate gene.

### 2.9. Statistical Analysis

The mRNA expression levels in patients with glioblastoma were compared by the Mann–Whitney *U* test between groups. Categorical variables were expressed as frequency and compared using the chi-square test. Correlation between mRNA expression and LNR was tested using Spearman's rank correlation test. The candidate gene expression level higher than the median value was considered high expression, whereas the gene expression level lower than the median value was considered low expression. The OS was presented in Kaplan–Meier curves. The Kaplan–Meier survival curves with the log-rank test were calculated and then plotted using GraphPad Prism version 8. *P* < 0.05 was considered to indicate a statistically significant difference.

## 3. Results

### 3.1. Patient Characteristics

For gene expression analysis, 33 glioblastoma patients who underwent preoperative ^18^F-FMISO/^18^F-FDG PET were included. As shown in Table 1, study participants consisted of 16 female (48.4%) and 17 male (51.6%) patients (age 61.0 ± 15.6 years). *IDH* wild type in 32 patients and *IDH1*-R132H mutation in one patient were confirmed with retained 1p19q.

### 3.2. The Relationship between Hypoxia- and Glucose-Related Gene Expression and FDG PET Value

**Figure 2:**
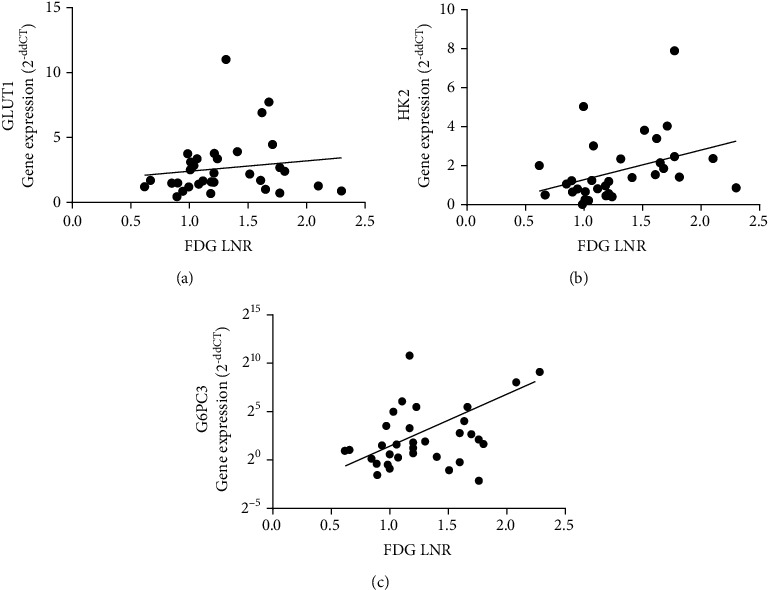
Relationship between glucose metabolism-related molecule mRNA levels and fluorine-18-fluorodeoxyglucose (^18^F-FDG) uptake lesion-to-normal brain ratio (LNR). (a) *Glucose transporter 1* (*GLUT1*) mRNA levels did not correlate with ^18^F-FDG LNR (*P* = 0.43). (b, c) Hexokinase 2 and glucose-6-phosphatase catalytic unit 3 mRNA levels significantly correlated with FDG LNR (*P* = 0.03, *Y* = 1.52 *X*–0.24, *R*^2^ = 0.14 and *P* = 0.02, log_2_Y = 3.02 *X*–1.40, *R*^2^ = 0.16, respectively). These were tested by Spearman's rank correlation coefficient.

The LNR of ^18^F-FDG uptake did not correlate with *GLUT1* mRNA expression in Figure 2(a) (*P* = 0.4271). Among the genes examined, *HK2* and *G6PC3* mRNA expressions were significantly correlated with ^18^F-FDG uptake (*HK2*: *P* = 0.03, *R*^2^ = 0.138; *G6PC3*: *P* = 0.021, *R*^2^ = 0.161) (Figures 2(b) and 2(c)). Other molecules including *G6PC1* mRNA expression were not correlated with ^18^F-FDG uptake (Figure S1).

### 3.3. Comparison between Hypoxia- and Glucose-Related Gene Expressions at the Intensity of FMISO Accumulation

**Figure 3:**
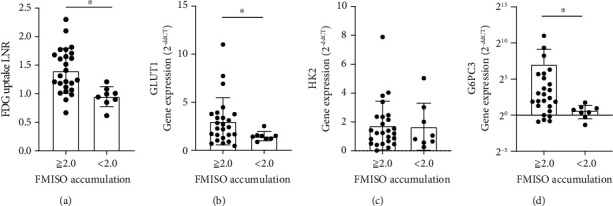
(a–d) ^18^F-fluorodeoxyglucose (^18^F-FDG) uptake LNR was higher in tissues with high ^18^F-fluoromisonidazole (^18^F-FMISO) accumulation than in tissues with low ^18^F-FMISO accumulation (*P* = 0.001). Comparison of mRNA levels with a threshold of ^18^F-FMISO accumulation: lesion-to-normal brain ratio (LNR) 2.0. mRNA levels of (b) *glucose transporter 1* (*GLUT1*) and (d) *glucose-6-phosphatase catalytic unit 3* (*G6PC3*) were higher in tissues with high ^18^F-FMISO accumulation than in tissues with low ^18^F-FMISO accumulation (*GLUT1*: *P* = 0.04; *G6PC3*: *P* < 0.01). mRNA expression of (c) *hexokinase 2* (*HK2*) remained unchanged depending on ^18^F-FMISO accumulation (*P* = 0.79). The statistical difference was calculated by using the Mann–Whitney *U*-test. ^∗^*P* < 0.05.

Next, we investigated the correlation between the mRNA expression of each gene and the intensity of FMISO accumulation. As described above, glioblastomas were divided into high and low FMISO accumulation in a 2.0 threshold of FMISO LNR. In this series, ^18^F-FDG uptake LNR in high ^18^F-FMISO-accumulated tissues was higher than in low ^18^F-FMISO-accumulated tissues (Figure 3(a)). mRNA levels of *GLUT1* and *G6PC3* in samples from patients with high ^18^F-FMISO accumulation were significantly higher than those from patients with low FMISO accumulation (*GLUT1*: *P* = 0.04; *G6PC3*: *P* < 0.01; Figures 3(b) and 3(c)). In contrast, there is no difference in *HK2* mRNA expression depending on ^18^F-FMISO accumulation (*P* = 0.79; Figure 3(d)). Other molecule mRNA expression did not have significant difference depending on ^18^F-FMISO accumulation (Figure S2).

### 3.4. The Correlation between G6PC3 Gene and Protein Expression in Glioblastoma

**Figure 4:**
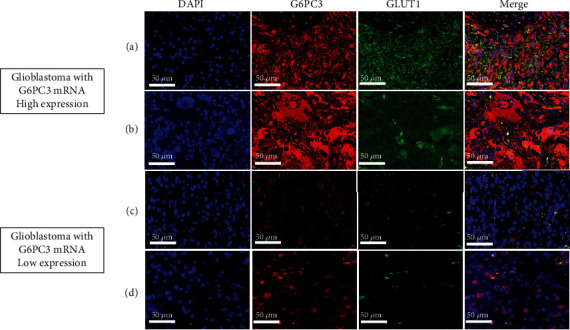
Immunofluorescence staining of glioblastoma using the anti-glucose-6-phosphatase catalytic unit 3 (G6PC3) and anti-glucose transporter 1 (GLUT1) antibody. Nuclei were stained with 4′,6-diamidino-2-phenylindole (DAPI). (a, b) Glioblastoma samples with high *G6PC3* mRNA expression showed G6PC3 and GLUT1 expression. (c, d) Glioblastoma samples with low *G6PC3* mRNA expression hardly showed their expression. Bars in panel = 50 *μ*m.

To confirm whether protein synthesis was regulated by mRNA regarding *G6PC3* expression, immunofluorescence staining was performed. As shown in Figure 4, merged images showed that G6PC3 was strongly expressed in cytoplasm of all samples with high mRNA expression while samples with G6PC3 low expression had faintly stained cytoplasm. Anti-GLUT1 staining had similar tendency of anti-G6PC3.

### 3.5. The Correlation between G6PC3 Expression and Overall Survival of Patients with Glioblastoma

**Figure 5:**
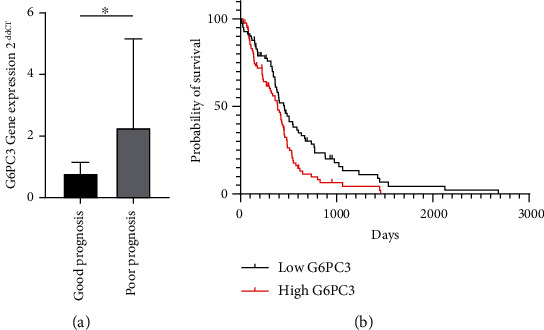
The significance of *G6PC3* mRNA expression on the prognosis. (a) In another cohort in our institute, *G6PC3* mRNA expression was significantly different between the two groups. Higher *G6PC3* mRNA expression was observed in the poor prognosis group than in the good prognosis group (good prognosis: median 0.88, *n* = 8; poor prognosis: median 1.40, *n* = 9; *P* < 0.05, Mann–Whitney test). (b) In The Cancer Genome Atlas database, two groups were stratified according to a threshold of the median *G6PC3* mRNA expression. Patients with higher *G6PC3* mRNA expression (*n* = 84, median 385.0 days, 95% C.I. 0.46–0.91) had poorer overall survival than those with lower mRNA expression (*n* = 83, median 455.0 days, 95% C.I. 1.11–2.18) by log-rank test (*P* = 0.008). 95% confidence interval: 95% C.I.

Since G6PC3 might be a key molecule of glioblastoma under hypoxic condition thorough previous evaluation, prognostic analysis in glioblastoma was performed whether G6PC3 expression is associated with the survival. First, using different cohorts in our archive, the *G6PC3* mRNA expression of glioblastoma tissues was investigated in two groups of patients according to their prognosis (Table. S2). This demonstrated that *G6PC3* mRNA expression was markedly higher in the poor prognosis group than that in the good prognosis group (*P* = 0.027) (Figure 5(a)). Then, we further attempted to validate the correlation between *G6PC3* gene expression and prognosis using TCGA database for a larger population (Table. S3). Patients with high *G6PC3* mRNA expression (*n* = 84, median 385.0 days, 95% C.I. 0.46–0.91) had significantly poorer OS than those with low *G6PC3* mRNA expression (*n* = 83, median 455.0 days, 95% C.I. 1.11–2.18) (*P* = 0.008) (Figure 5(b)). These results suggested that high *G6PC3* expression is associated with poor prognosis in glioblastoma.

## 4. Discussion

Using ^18^F-FDG and ^18^F-FMISO PET, we previously demonstrated that the degree of glucose metabolic activation in hypoxic area in glioblastoma was correlated their prognosis [9]. In this exploratory study, we identified the potential key molecule, G6PC3, to highly activate glucose metabolism under hypoxia. G6PC3 expression was elevated in tissues of glioblastoma with higher ^18^F-FDG and ^18^F-FMISO accumulation, and also, *G6PC3* elevation was related to the poor prognosis in another independent cohort as well as TCGA database, indicating that it could become a prognostic marker in glioblastomas.

In malignant tumor cells, especially under severe hypoxic conditions, HIF-1*α* is often promoted through signal transduction pathways, such as the phosphoinositide-3-kinase/Akt/mTOR pathway, extracellular-regulated protein kinase pathway, and adenosine 5′-monophosphate-activated protein kinase pathway. Hypoxia-induced metabolic reprogramming was essential to satisfy cellular energetic demands during acute hypoxic stress via HIF-1*α* [25, 26]. Tumor cells reduce their dependency on mitochondrial oxidative phosphorylation and switch to the O_2_-independent glycolytic pathway to promote adenosine triphosphate production to meet their demands [17]. HIF-1*α* forms a complex of HIF-1*α* or other proteins and adheres to hypoxia-responsive elements (HRE; i.e., 5′-ACGTG-3′) on the genome line. HIF-1*α* binds with HRE and stimulates the downstream enzymes in the glycolytic system, such as glucose transporters, hexokinase (HK), pyruvate kinase, and lactate dehydrogenase A [26, 27]. This cascade of response to hypoxia enables tumor cells to metabolize glucose anaerobically and survive under severe hypoxia.

We started this study with a hypothesis that molecular features relating to ^18^F-FDG and ^18^F-FMISO accumulation might affect tumor aggressivity. According to previous investigations, ^18^F-FDG uptake has been reported to reflect GLUT1 [28, 29] and HK2 [30, 31] in gliomas or other malignant tumors. Therefore, we determined molecules associated with glucose and investigate the relationship between mRNA expression and a lesional semiquantitative value obtained from ^18^F-FDG and ^18^F-FMISO PET. We could not identify the single parameter as an ^18^F-FDG uptake regulator. Not only *GLUT1* but also *HK2* and *G6PC3* were significantly correlated with ^18^F-FDG uptake, although *GLUT1* also tended to correlate with ^18^F-FDG uptake. This was partially not consistent with that of previous studies that reduced G6Pase exhibited a high ^18^F-FDG uptake in some tumors [32]. Since glucose metabolism might be determined by multiple factors in complex manners, the exact mechanism remains unclear.

In previous reports, G6Pase was thought to be associated with ^18^F-FDG6P washout [13, 16, 33]. Generally, it was reported to be inversely correlated with ^18^F-FDG uptake in hepatocellular carcinoma [34]. However, a recent analysis for ^18^F-FDG kinetics delineated that ^18^F-FDG-6P hydrolysis by G6Pase was extremely low to be negligible, whereas ^18^F-FDG uptake and glucose consumption were tremendously high [33, 35]. Furthermore, ^18^F-FDG release has been found to reflect the expression of the glucose-6-phosphate transporter more than that of G6Pase [13, 14]. Altogether, G6Pase did not function as a negative factor of ^18^F-FDG uptake since the glycolysis metabolites might shunt into gluconeogenesis via G6Pase due to increased ^18^F-FDG uptake. Although G6PC3 has been less likely known to correlate with ^18^F-FDG uptake in glioma, a study reported that the maximum SUV of ^18^F-FDG uptake correlated with *G6PC3* mRNA expression in intrahepatic cholangiocarcinoma [36].

GLUT1 and G6PC3 expressions were significantly correlated with high ^18^F-FMISO accumulation in this study. GLUT1 was a well-known molecule induced by hypoxia [17, 37]. G6Pase was also reported to be increased under hypoxia by the HIF-1*α* activation [38]. Conversely, G6PC3 was not significantly changed by hypoxia in the mesenchymal stem cells [39]. No study has reported the effects of hypoxia on *G6PC3* mRNA expression in glioma. Therefore, the influence of hypoxia on G6PC3 expression should be further explored.

G6Pase catalyzes the hydrolysis to glucose or ^18^F-FDG-6-phosphate to ^18^F-FDG. It is located in the endoplasmic reticulum [16, 40]. G6Pase was reported to play a protumorigenic role in cell proliferation and migration mainly by regulating oncogenic signaling in glioblastoma. Starving brain tumor-initiating cell was able to survive under glycolytic inhibition, owing to the function of G6Pase as a gluconeogenesis catalyst. Interestingly, surviving cells had more aggressive characteristics after glycolytic inhibition in terms of migration, invasion, and proliferation [41]. G6PC3 is an isoform of G6Pase and was originally known as G6Pase-*β*. The G6Pase gene family consists of three types of isoforms: G6PC, G6PC2, and G6PC3. G6PC3 is expressed ubiquitously although predominantly in the brain, muscle, and kidney [16, 42]. In our study, G6PC3 protein synthesis was found to be consistent with mRNA expression in glioblastoma. Given that GLUT1 expression had similar tendency, G6PC3 might have an important role in glucose metabolism affecting tumor aggressivity.

Lastly, prognostic analysis was performed using two independent patient cohorts. Both of them proved that higher *G6PC3* mRNA expression was associated with a poorer prognosis based on OS. In particular, the Kaplan–Meier survival curve analysis in TCGA dataset revealed that high *G6PC3* expression was significantly associated with poor OS. Recently, *G6PC3* has been focused on as a candidate prognostic predictor of glioblastoma through big data gene expression analysis [43]. However, no reports have elucidated the molecular mechanism of the relationship between the *G6PC3* gene expression and prognosis. Then, G6PC3 induction by hypoxia can be its clue.

Regarding the study limitations, first, mRNA gene expression of frozen samples should be interpreted with tumor heterogeneity. The validity of the tumor sampling site was difficult. Second, the number of samples was small. Although this study could not unravel the underlying mechanism, G6PC3 was found to be a potential candidate of potent prognostic factor.

## 5. Conclusions

G6PC3 was affluently expressed in the tumor with high ^18^F-FDG and ^18^F-FMISO accumulation. It might be a key molecule of glucose metabolism under hypoxia in glioblastoma. Moreover, it can also work as a prognostic predictor of glioblastoma.

## Figures and Tables

**Table 1:** Patient characteristics and the relationship between mRNA expression and clinicopathological characteristics in glioblastoma patients who preoperatively underwent fluorine-18-fluorodeoxyglucose/fluorine-18-fluoromisonidazole PET.

Parameters	No. of patients (%)	
Age (years)	Median (±SD)	61.0 (±15.6)
Gender	Female	16
	Male	17
Gene status	IDH wild type	32
	IDH mutant without 1p/19q codeletion	1
Histology	Glioblastoma (grade IV)	33

KPS: Karnofsky performance score; IDH: isocitrate dehydrogenase.

## Data Availability

The datasets used and/or analyzed during the current study are available from the corresponding author on reasonable request.

## Abbreviations

CT: Computed tomography
^18^F-FMISO: Fluorine-18-fluoromisonidazole
^18^F-FDG: Fluorine-18^−^-fluorodeoxyglucose
FFPE: Formalin-fixed paraffin-embedded
GLUT: Glucose transporter
G6Pase: Glucose-6-phosphatase
G6PC: Glucose-6-phosphatase catalytic unit
HIF: Hypoxia-inducible factor
HK: Hexokinase
HRE: Hypoxia-responsive elements
IDH: Isocitrate dehydrogenase
LNR: Lesion-normal tissue ratio
OS: Overall survival
PCNA: Proliferation cell nuclear antigen
RT-qPCR: Reverse transcriptase quantitative polymerase chain reaction
SUV: Standardized uptake value
TCGA: The Cancer Genome Atlas
VEGF: Vascular endothelial growth factor
WHO: World Health Organization.

## Supplementary Materials

Supplementary 1 Figure S1: relationship between glucose metabolism associated with mRNA levels and ^18^F-FDG uptake LNR. (a) *Glucose transporter 3* (*GLUT3*), (b) *glucose-6-phosphatase catalytic subunit 1* (*G6PC1*), (c) *G6PC2*, (d) *hexokinase 1* (*HK1*), (e) *vascular endothelial growth factor* (*VEGF*), and (f) *proliferation cell nuclear antigen* (*PCNA*) mRNA levels were not correlated with ^18^F-FDG uptake LNR.

Supplementary 2 Figure S2: comparison of mRNA levels with a threshold of ^18^F-FMISO accumulation; LNR 2.0. (a) *Glucose transporter 3* (*GLUT3*), (b) *glucose-6-phosphatase catalytic subunit 1* (*G6PC1*), (c) *G6PC2*, (d) *hexokinase 1* (*HK1*), (e) *vascular endothelial growth factor* (*VEGF*), and (f) *proliferation cell nuclear antigen* (*PCNA*) mRNA levels were changed between ^18^F-FMISO accumulation.

Supplementary 3 Table S1: the primer sequences of *GLUT1*, *GLUT3*, *G6PC1*, *G6PC2*, *G6PC3*, *HK1*, *HK2*, *PCNA*, *VEGF*, and *18S rRNA*.

Supplementary 4 Table S2: patient characteristics and the relationship between *G6PC3* mRNA expression and clinicopathological characteristics in glioblastoma patients in another cohort for validation.

Supplementary 5 Table S3: patient characteristics and the relationship between G6PC3 mRNA expression and clinicopathological characteristics in glioblastoma patients based on the TCGA database.
