# Supplementary figures and images for: Identifying G6PC3 as a Potential Key Molecule in Hypoxic Glucose Metabolism of Glioblastoma Derived from the Depiction of 18F-Fluoromisonidazole and 18F-Fluorodeoxyglucose Positron Emission Tomography
Source: Biomed Res Int. 2024 Feb 28;2024:2973407. doi: 10.1155/2024/2973407 (PMC10917478; doi:10.1155/2024/2973407)

Figure. S1

(a)

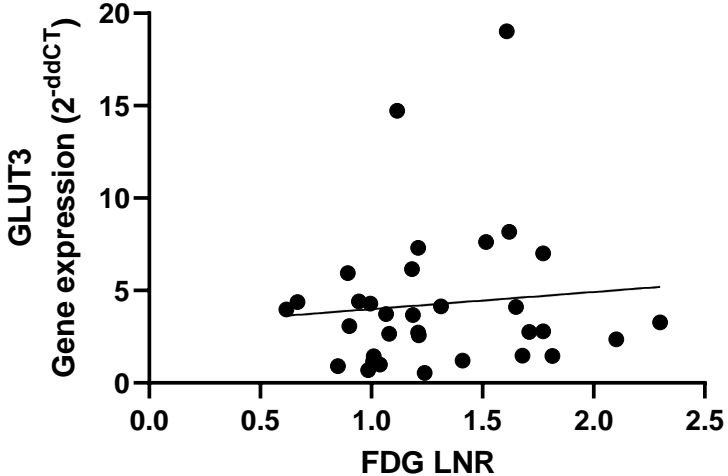

(b)

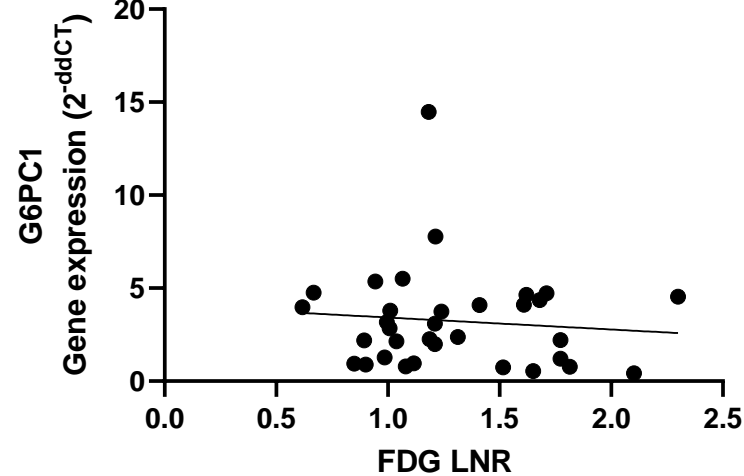

(c)

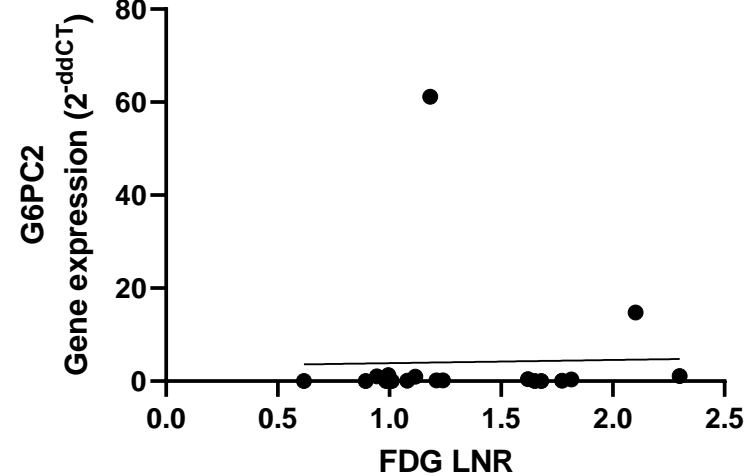

(d)

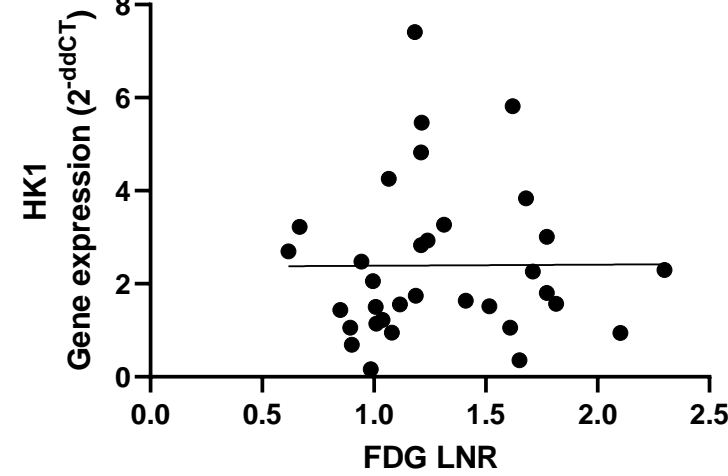

(e)

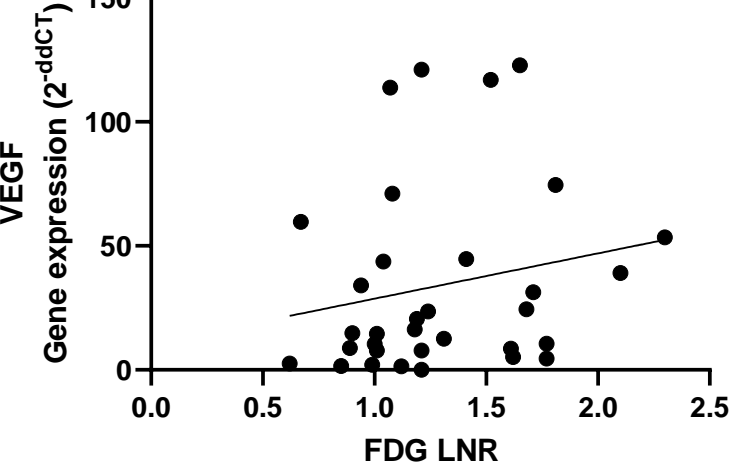

(f)

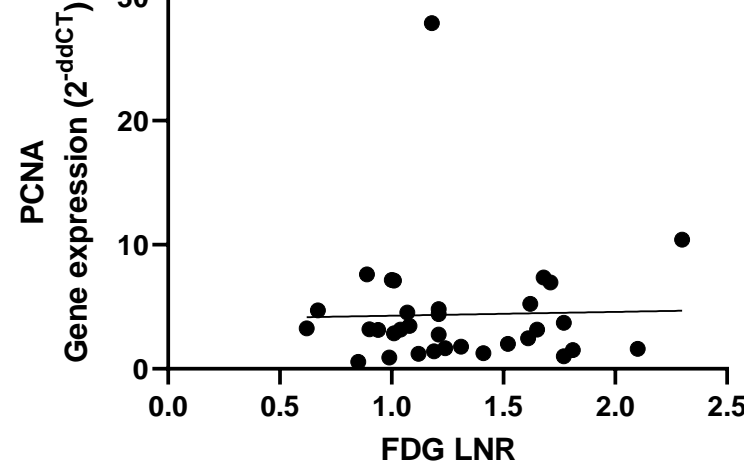

Supplement: Supplementary 1 — Figure S1: relationship between glucose metabolism associated with mRNA levels and 18F-FDG uptake LNR. (a) Glucose transporter 3 (GLUT3), (b) glucose-6-phosphatase catalytic subunit 1 (G6PC1), (c) G6PC2, (d) hexokinase 1 (HK1), (e) vascular endothelial growth factor (VEGF), and (f) proliferation cell nuclear antigen (PCNA) mRNA levels were not correlated with 18F-FDG uptake LNR. [file 2973407.f1.pdf]

Figure. S2

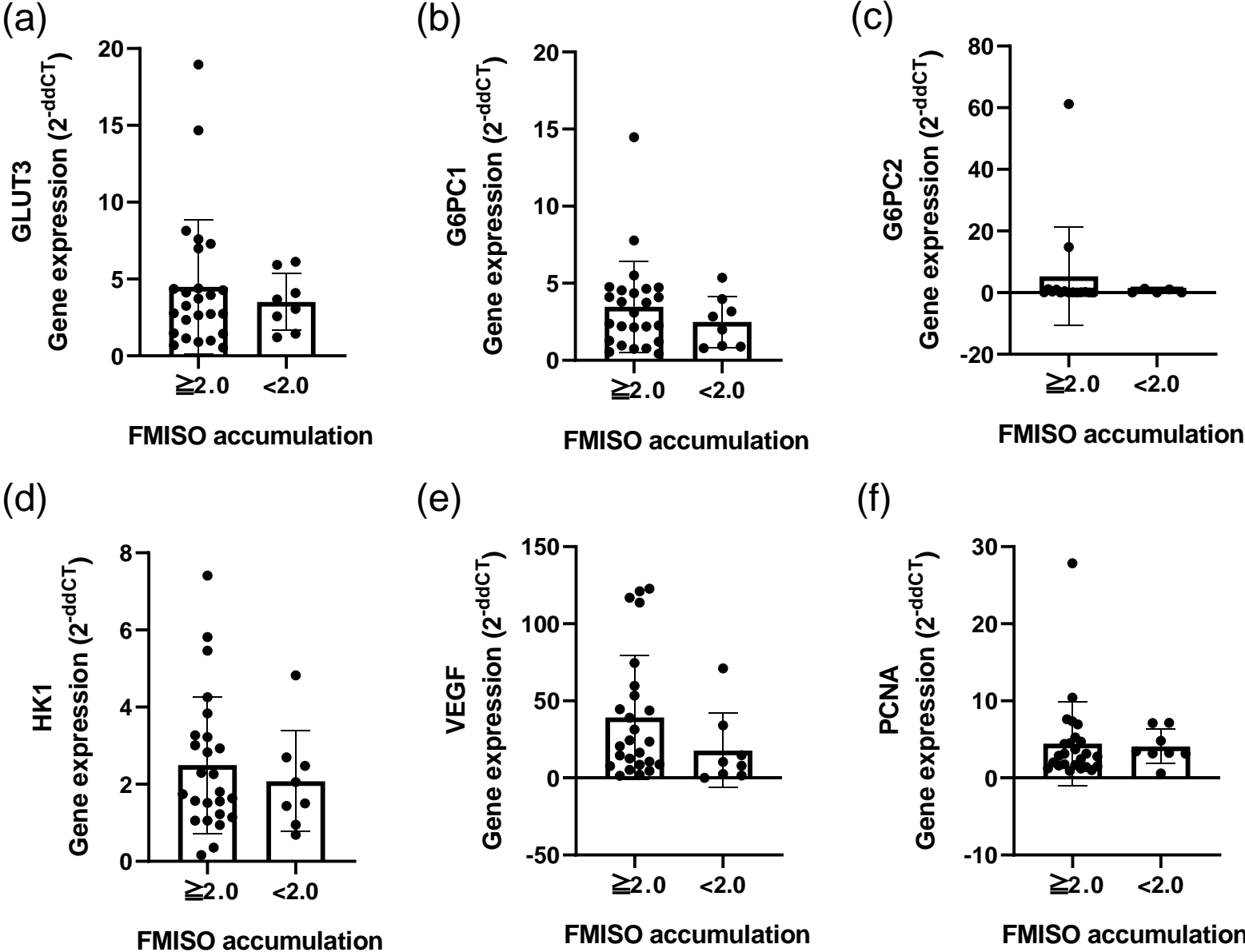

Supplement: Supplementary 2 — Figure S2: comparison of mRNA levels with a threshold of 18F-FMISO accumulation; LNR 2.0. (a) Glucose transporter 3 (GLUT3), (b) glucose-6-phosphatase catalytic subunit 1 (G6PC1), (c) G6PC2, (d) hexokinase 1 (HK1), (e) vascular endothelial growth factor (VEGF), and (f) proliferation cell nuclear antigen (PCNA) mRNA levels were changed between 18F-FMISO accumulation. [file 2973407.f2.pdf]
