# Supplementary material for: Identifying G6PC3 as a Potential Key Molecule in Hypoxic Glucose Metabolism of Glioblastoma Derived from the Depiction of 18F-Fluoromisonidazole and 18F-Fluorodeoxyglucose Positron Emission Tomography
Source: Biomed Res Int. 2024 Feb 28;2024:2973407. doi: 10.1155/2024/2973407 (PMC10917478; doi:10.1155/2024/2973407)
Supplement: Supplementary 3 — Table S1: the primer sequences of GLUT1, GLUT3, G6PC1, G6PC2, G6PC3, HK1, HK2, PCNA, VEGF, and 18S rRNA. [file 2973407.f3.doc]

**Table S1. The primer sequences of *GLUT1, GLUT3, G6PC1, G6PC2, G6PC3, HK1, HK2, PCNA, VEGF,* and *18S rRNA.***

| Gene | Forward Primer (5’-3’) | Reverse Primer (3’-5’) |
| --- | --- | --- |
| *GLUT1* | CCTTTTCGTTAACCGCTTTGG | CAGGATCAGCATCTCAAAGGACTT |
| *GLUT3* | CGTGGAGAAAACTTGCTGCTG | ATCAGAGCTGGGGTGACCTTCT |
| *G6PC1* | GATAAAGCCGACCTACAGATTTCG | GCAGCAAGGTAGATTCGTGACA |
| *G6PC2* | TGTGAAACAGGTCCAGGCAT | TGAGCACCCTAAGAAGCAGG |
| *G6PC3* | TCTTCAAGTGGTTTCTTTTTGGAG | GCTAGGCATCACCCTTACCC |
| *HK1* | GCGATGACAGTATCCTCGTCAA | TCTCGCGGATCTTATCCACAA |
| *HK2* | ACCCAGTTCATTCACATCATCAGT | CCCTCTACTTTAAGGCCCTATTCG |
| *PCNA* | GAAGGTGTTGGAGGCACTCAA | AGGGTGAGCTGCACCAAAGA |
| *VEGF* | CTACCTCCACCATGCCAAGT | GCAGTAGCTGCGCTGATAGA |
| *18S rRNA* | CGAACGTCTGCCCTATCAACTT | ACCCGTGGTCACCATGGTA |
