# Supplementary material for: Identifying G6PC3 as a Potential Key Molecule in Hypoxic Glucose Metabolism of Glioblastoma Derived from the Depiction of 18F-Fluoromisonidazole and 18F-Fluorodeoxyglucose Positron Emission Tomography
Source: Biomed Res Int. 2024 Feb 28;2024:2973407. doi: 10.1155/2024/2973407 (PMC10917478; doi:10.1155/2024/2973407)
Supplement: Supplementary 4 — Table S2: patient characteristics and the relationship between G6PC3 mRNA expression and clinicopathological characteristics in glioblastoma patients in another cohort for validation. [file 2973407.f4.doc]

**Table S2.** Patient characteristics and the relationship between *G6PC3* mRNA expression and clinicopathological characteristics in glioblastoma patients in another cohort for validation.

| **Parameters** | **No. of patients (%)** | **Prognosis** | | **P value** |
| --- | --- | --- | --- | --- |
|  |  | **good (n=8)** | **poor (n=9)** |  |
| **Age (years)**  ≤60  >60 | 3  14 | 66.0 (±11.5)  1  7 | 66.0 (±10.1)  2  7 | 0.5997 |
| **Gender**  Female  Male | 5  12 | 2  7 | 3  5 | 0.4902 |
| **KPS**  ≥70  <70 | 11  6 | 82.5 (±7.1)  7  1 | 67.8 (±17.9)  4  5 | 0.0637 |
| **IDH status**  IDH wild type  IDH mutant | 17  0 | 8  0 | 9  0 | - |
| **Adjuvant radiochemotherapy**  Yes  No | 17  0 | 8  0 | 9  0 | - |
| **Last status**  Alive  Dead | 1  16 | 1  7 | 0  9 | 0.4706 |
| **Overall survival**  Median (days)  95% C.I |  | 1590  0.065 - 0.470 | 278  2.130 - 15.36 | 0.0001 |

KPS, Karnofsky performance score

IDH, isocitrate dehydrogenase

95% C.I, 95% confidence interval
