# Supplementary material for: Identifying G6PC3 as a Potential Key Molecule in Hypoxic Glucose Metabolism of Glioblastoma Derived from the Depiction of 18F-Fluoromisonidazole and 18F-Fluorodeoxyglucose Positron Emission Tomography
Source: Biomed Res Int. 2024 Feb 28;2024:2973407. doi: 10.1155/2024/2973407 (PMC10917478; doi:10.1155/2024/2973407)
Supplement: Supplementary 5 — Table S3: patient characteristics and the relationship between G6PC3 mRNA expression and clinicopathological characteristics in glioblastoma patients based on the TCGA database. [file 2973407.f5.doc]

**Table S3.** Patient characteristics and the relationship between G6PC3 mRNA expression and clinicopathological characteristics in glioblastoma patients based on the TCGA database.

| **Parameters** | **No. of patients (%)** | **G6PC3 expression** | | **P value** |
| --- | --- | --- | --- | --- |
|  |  | **High (n=84) Low (n=83)** | |  |
| **Age (years)**  ≤60  >60 | 79  88 | 33  51 | 46  37 | 0.1035 |
| **Gender**  Female  Male | 59  108 | 34  50 | 25  58 | 0.1616 |
| **KPS**  ≥70  <70  Unknown | 91  33  43 | 50  15  19 | 41  18  24 | 0.3497 |
| **IDH status**  IDH wild type  IDH mutant  Unknown | 137  9  21 | 75  3  6 | 62  6  15 | 0.2123 |
| **EOR**  Tumor resection  Biopsy | 152  16 | 76  8 | 75  8 | 0.9799 |
| **Adjuvant radiotherapy**  Yes  No  Unknown | 138  21  8 | 68  10  6 | 70  11  2 | 0.8875 |
| **Adjuvant chemotherapy**  Yes  No  Unknown | 134  23  10 | 68  10  6 | 66  13  4 | 0.5195 |
| **Last status**  alive  dead | 31  136 | 12  72 | 19  64 | 0.1527 |

KPS, Karnofsky performance score

IDH, isocitrate dehydrogenase

EOR, extent of surgical resection
